# Regulation of Immune Responses by the Neonatal Fc Receptor and Its Therapeutic Implications

**DOI:** 10.3389/fimmu.2014.00664

**Published:** 2015-01-05

**Authors:** Timo Rath, Kristi Baker, Michal Pyzik, Richard S. Blumberg

**Affiliations:** ^1^Department of Medicine, Division of Gastroenterology, Brigham and Women’s Hospital, Harvard Medical School, Boston, MA, USA; ^2^Department of Medicine, Division of Gastroenterology, Erlangen University Hospital, Friedrich Alexander University Erlangen-Nueremberg, Erlangen, Germany; ^3^Harvard Digestive Diseases Center, Boston, MA, USA

**Keywords:** neonatal Fc receptor, immunoglobulin G, albumin, mucosal immunology, antigen presentation

## Abstract

As a single receptor, the neonatal Fc receptor (FcRn) is critically involved in regulating albumin and IgG serum concentrations by protecting these two ligands from degradation. In addition to these essential homeostatic functions, FcRn possesses important functions in regulating immune responses that are equally as critical and are increasingly coming to attention. During the first stages of life, FcRn mediates the passive transfer of IgG across the maternal placenta or neonatal intestinal walls of mammals, thereby conferring passive immunity to the offspring before and after birth. In fact, FcRn is one of the very few molecules that are known to move from luminal to serosal membranes of polarized cells that form epithelial barriers of the lung and intestines. Together with FcRn’s recently explored critical role in eliciting MHC II presentation and MHC class I cross-presentation of IgG-complexed antigen, this renders FcRn capable of exerting broad and potent functions in regulating immune responses and immunosurveillance at mucosal sites. Further, it is now clear that FcRn dependent mucosal absorption of therapeutic molecules is a clinically feasible and potent novel route of non-invasive drug delivery, and the interaction between FcRn and IgG has also been utilized for the acquisition of humoral immunity at mucosal sites. In this review, we begin by briefly summarizing the basic knowledge on FcRn expression and IgG binding, then describe more recent discoveries pertaining to the mechanisms by which FcRn orchestrates IgG related mucosal immune responses and immunosurveillance at host–environment interfaces within the adult organism. Finally, we outline how the knowledge of actions of FcRn at mucosal boundaries can be capitalized for the development and engineering of powerful mucosal vaccination strategies and novel routes for the non-invasive delivery of Fc-based therapeutics.

## FcRn Binds IgG Molecules Throughout Adult Life

FcRn, encoded by the *Fcgrt* gene, is an MHC class I-like transmembrane protein that requires non-covalent association with β2-microglobulin (β2-m) for proper functioning. In its best known function, FcRn binds to the CH2-CH3 interface of the IgG Fc region in a 2:1 stoichiometry (1–3). Binding between IgG and FcRn occurs in a strictly pH-dependent manner with micro- to nanomolar affinity at pH 6.5 while binding is virtually absent at pH 7.5 (4, 5). As revealed by site-directed mutagenesis, the residues Ile253, His310, and His435 of IgG are critically involved in its interaction with FcRn (4, 6–8). Consistent with such strict pH-dependent binding, the bulk of FcRn is expressed intracellularly – predominantly in endosomes – and such that the interaction between FcRn and its ligands occurs within an intracellular acidic milieu (9–12). Although originally viewed as a receptor restricted to neo- and antenatal life, it is now clear that FcRn continues to function throughout adult life and is expressed lifelong in both parenchymal cells (epithelium of the lung and intestine as well as the liver and vascular endothelium) and hematopoietic cells [monocytes, macrophages, dendritic cells (DCs), polymorphonuclear leukocytes, and B cells] in mouse and humans [as reviewed elsewhere (13–17)]. Apart from regulating and extending the serum half-life of IgG by a mechanism that is operative in DCs and endothelial cells (18–21), FcRn orchestrates IgG-based immune responses at mucosal sites. Collectively, the numerous functions of FcRn enable it to act as a sensitive regulator of mucosal immunity via bidirectional transcytosis of IgG and luminal antigens across epithelial boundaries as well as the active induction of MHC II presentation and MHC I cross-presentation pathways for the generation of antigen-specific T-cell responses.

## Mucosal Immune Regulation and Immunosurveillance by FcRn

**Figure 1:**
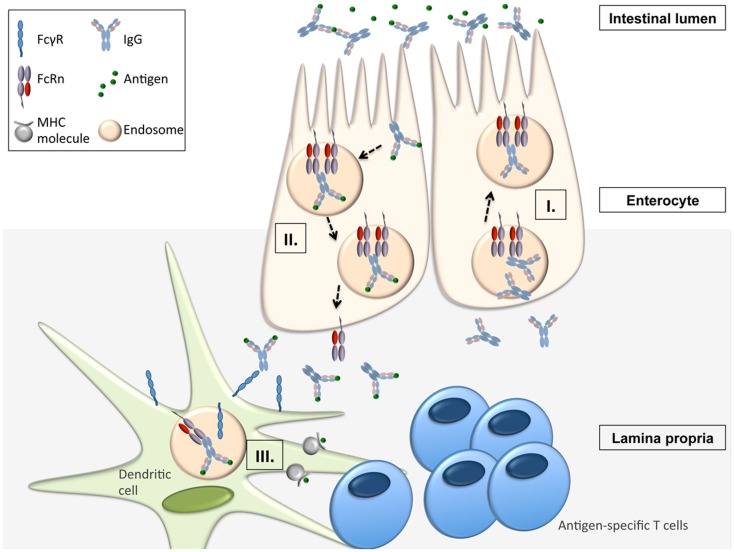
**FcRn-mediated functions in immune surveillance and homeostasis at the intestinal epithelial barrier (16, 31, 34)**. FcRn within enterocytes can transport IgG from the systemic circulation across the epithelial barrier into the intestinal lumen (I.). Upon formation of immune complexes (ICs) consisting of bacteria and anti-bacterial IgG within the intestinal lumen, FcRn can transcytose these back into the LP (II.). In the LP, the ICs are delivered to antigen-presenting cells (APCs) from which the ICs are taken up via canonical FcγR’s. Once internalized into the cell, the ICs are then directed into acidic endosomes, in which FcRn is operative to mediate the presentation of IC derived antigen to antigen-specific T-cells (III.).

Apart from being responsible for the transplacental transfer of maternal IgG to a human fetus, FcRn has been shown to mediate the bidirectional transport of IgG across all polarized epithelial barriers including those of the gastrointestinal and respiratory tract, the placenta, and the genitourinary system including kidneys (8, 22–29). Further, it has been documented that this mechanism is operative not only in humans and mice but also in rats, pigs, and non-human primates, implying that the transepithelial IgG transport across mucosal membranes is an evolutionarily well conserved and thus important part of immune regulation. By expressing FcRn, the epithelial barrier thus gains the ability to transport IgG from the basolateral to the apical side and vice versa, or, in other words, to deliver IgG into the lumen from the tissue space and, subsequently, transport IgG-bound luminal antigens back to the lamina propria (LP). This process is of utmost importance for immune regulation at mucosal barriers and *in vivo* studies have impressively demonstrated that FcRn is indeed a key molecule in integrating humoral and cellular immunity and intraluminal (and thereby foreign) signals into the host intramural immune system. To mirror the situation in humans, these studies utilized humanized mice in which a human FcRn transgene (together with human β2-m) was expressed in a mouse that was devoid of mouse FcRn (30, 31). *In vivo* studies using the model antigen ovalbumin (OVA) demonstrated that after intravenous injection of anti-OVA IgG and subsequent oral feeding of fluorescently labeled OVA, OVA-IgG immune complexes (ICs) formed within the gastrointestinal lumen and were transported in an FcRn dependent manner into the LP where the antigen–antibody complex was taken up by CD11c^+^ DCs which, upon MHC II presentation of OVA epitopes, were then able to induce an antigen-specific CD4^+^ T-cell response (31). Of note, the mechanism of FcRn-mediated transfer of antigen-specific IgG and subsequent induction of an antigen-specific T-cell response was not limited to the intestinal tract, as nasal administration of OVA after intravenous injection of anti-OVA IgG led to a similar FcRn-mediated uptake of antigen–antibody ICs and an expansion of antigen-specific CD4^+^ T-cells in the nasal-associated lymphatic tissue (31). This mechanism has direct physiologic and immunopathogenic relevance: when challenged with *Citrobacter rodentium* – which models enteropathogenic *Escherichia coli* infection – mice with restricted expression of FcRn solely within the intestinal epithelium, but not FcRn deficient animals, were protected from infection but only in the presence of anti-*C. rodentium* IgG antibodies, consistent with IgG being a main effector molecule in the eradication of this pathogen (32–34). To mount an effective immune response against this epithelial pathogen, FcRn was shown to mediate the following processes (Figure 1): (I.) transport of pathogen-specific IgG from the systemic circulation across the epithelial barrier into the intestinal lumen, (II.) uptake of the newly formed ICs consisting of bacteria and anti-bacterial IgG and transcytosis of these into the LP, and (III.) induction of an antigen-specific immune response and T-cell expansion within regional lymphoid structures and associated peripheral tissues (34).

Thus, these studies provide direct evidence that epithelial expression of FcRn is able to link luminal and/or epithelial infectious exposures with systemic immune activation. FcRn expression in the gastric epithelium and the associated transport of pathogen-specific IgG across the gastric epithelium is also linked to the prevention of gastric colonization and invasion by the pathogens *Helicobacter heilmannii* and *Helicobacter pylori* (35). Similarly, FcRn within the genitourinary and respiratory tract enables the transmucosal transport of pathogen-specific IgG and has been associated with the prevention of viral infection, as shown in model studies mirroring herpes simplex 2 or influenza infection (24, 29).

## FcRn’s Role in Antigen Presentation by Professional Antigen-Presenting Cells at Mucosal Surfaces

### Classical MHC II presentation of immune complexed antigen

While the later studies mainly investigated FcRn’s function of providing passive immunity by transcytosing IgG across the mucosal barrier, the discoveries that FcRn within the DC is deeply involved in the generation of antigen-specific T-cell responses opened up another dimension in FcRn’s pleiotropic functions for immune homeostasis. One critical prerequisite for addressing FcRn’s role in antigen presentation is to carefully control IgG levels. Both hematopoietic cells and parenchymal cells are equally responsible for protecting IgG from degradation (18–21, 30), and in order to account for IgG deficiency in *Fcgrt*^−/−^ mice, the utilization of bone-marrow chimeric mice is an elegant approach to circumvent differences in IgG levels between WT and *Fcgrt*^−/−^ mice, as previously shown (18, 19, 21).

After having shown that FcRn is expressed in professional APC in human and mouse (18, 19, 21, 31, 36–38), it was demonstrated that FcRn in DCs enhances MHC II antigen presentation and induces proliferation of antigen-specific CD4^+^ T-cells when antigen is presented as a multimeric IC with IgG, both *in vitro* and *in vivo* (21), and this observation was also confirmed in humans (39). Furthermore, both murine and human studies have demonstrated that ICs containing a non-FcRn-binding IHH-IgG mutant, which harbors three mutations that disable binding to FcRn (I253A, H435A, and H436A) (7), do not lead to T-cell activation (21). Importantly, the studies showed that this regulation is only observed when the IgG–antigen complex is provided as a multimeric IC, suggesting that FcRn directs the intracellular trafficking of its ligands differently depending on the valency of the bound ligands. This concept was supported by confocal microscopy showing that multimeric IgG ICs, but not IHH-IgG1 ICs or monomeric ICs, were rapidly internalized by human DCs and co-localized with FcRn first in membrane-proximal intracellular compartments, presumably early endosomes, and subsequently in LAMP1-positive lysosomes, thus indicating that FcRn directs multimeric ICs into a cellular compartment equipped with the machinery to induce MHC II antigen presentation (39). Using bone-marrow chimeric mice, it was then directly shown that the fate and also the associated serum half-life are indeed fundamentally different depending on the ligand valency. IgG present in multimeric ICs exhibited rapidly increased degradation mediated by hematopoietic cells, presumably DCs, consistent with an *in vivo* fate of active trafficking toward lysosomal degradation rather than FcRn-mediated recycling. However, unbound IgG or IgG in monomeric complexes were protected from degradation in an FcRn dependent manner from both hematopoietic and myeloid cells (39). Thus, these studies show that FcRn determines the fate of IgG depending on the nature of the ligand. In a more general sense, these findings illustrate that FcRn actively senses whether an antigen has already been IgG opsonized, in which case the IC are routed towards antigen presentation compartments, or whether IgG is unbound and thus should be protected from degradation.

### MHC I cross-presentation of immune complexed antigen

Recent studies further extend the understanding of the critical involvement of FcRn in antigen presentation at mucosal surfaces. Strikingly, CD8^−^CD11b^+^ DCs loaded with IgG-complexed OVA induced robust proliferation of adoptively transferred OT-I T-cells in an entirely FcRn-dependent manner (36). These studies were the first to discover that FcRn exhibits also a fundamental role in the presentation of exogenous antigen to CD8^+^ T-cells, a process called cross-presentation (36, 40). These studies also demonstrated that FcRn within CD8^−^CD11b^+^ DCs directed the IgG ICs into a Rab27a, vacuolar ATPase, and gp91phox containing acidic phagosome that allowed for cross-presentation (36). Further, utilizing antibodies that have no affinity for FcRn, but intact affinity for canonical FcγRs (IHH-IgG) or which cannot bind FcγRs, but retain the ability to bind FcRn [N297A-IgG (41)], it was shown that canonical FcγRs on the surface of DCs mediate the entry of the ICs into the cell which then are further directed and trafficked in an FcRn dependent fashion as outlined above (36). Thus, FcRn and canonical surface FcγR’s cooperate in inducing antigen presentation and the pH-dependent ligand binding of FcRn and its expression within the acidic milieu of endosomal compartments is perfectly suited to further handle, traffic and process IgG and ICs once bound at neutral pH by FcγRs and internalized.

The mechanisms and pathophysiological significance of FcRn dependent cross-presentation to DCs at mucosal surfaces has recently been analyzed (37). First, these studies demonstrated that untreated *Fcgrt*^−/−^ mice exhibit decreased numbers of CD8^+^ T-cells within the LP of the large intestine, and that these were also deficient in cytokine production. The utilization of *CD11c^Cre^Fcgrt^Fl/Fl^* mice (37) has shown that FcRn expression in DCs is critical for this. Second, FcRn within DCs was not only shown to be required for homeostatic CD8^+^ T-cell activation but also conferred protection from cancer development at mucosal sites, namely, the large intestine and the lung. This was reliant on the FcRn dependent induction of endogenous CD8^+^ T-cells towards cognate tumor antigens, and thus, these studies were the first to identify a deep involvement of FcRn in the protection from cancers arising at mucosal tissues. As an additional mechanism behind these observations, cross-linking of FcRn by IgG IC induced secretion of the cytotoxicity-promoting cytokine IL-12, thereby providing an additional stimulus to activate antigenically primed specific CD8^+^ T-cells (37). Importantly, analysis of the overall microbial community composition and diversity from wildtype and *Fcgrt*^−/−^ littermates revealed no significant differences in either post-weaning, 8-week-old mice or pre-weaning, 2-week-old mice in any of three separate intestine-associated tissue compartments, thereby confirming that the induction of mucosal CD8^+^ T-cells in FcRn bearing mice was not due to changes in the intestinal microbiota (37).

These findings are directly translatable to human pathology such that human DCs expressing high levels of FcRn co-localize with CD8^+^ T-cells in the stroma of both normal and colorectal cancer (CRC) large intestine and induce IL-12 production in an FcRn-dependent manner. Further, survival analysis indicated that CRC patients with ≥10 FcRn^+^CD11c^+^ cells per observed area had significantly longer survival times over a 70-month follow up than did those with <10 FcRn^+^CD11c^+^ cells in the tumor microenvironment (37). Finally, this mechanism was shown to be amenable to therapeutic manipulation. When ICs were formed with an engineered IgG variant known to exhibit enhanced FcRn binding with maintained pH dependency (42), antigen-specific CD8^+^ T-cell activation was induced at antigen concentrations 10-fold lower than that observed with native IgG IC, thereby demonstrating that targeting the immunostimulatory potential of FcRn using complexes formed with IgG variants having increased FcRn binding (15) and which are restricted to a single defined tumor antigen is a tractable and effective anti-tumor therapeutic approach (37). Further, proof-of-principle experiments demonstrated that *ex vivo* priming of CD8^+^ T-cells towards a single FcRn-targeted antigen expressed by tumor cells enables subsequent *in vivo* protection from the seeding of lung metastases. These studies demonstrate the potential of therapeutically promoting tumor immune surveillance in healthy, high-risk individuals by enhancing the baseline cytotoxic potential of the intestine by targeting and modulating FcRn binding to IgG (37).

## FcRn Dependent Non-Invasive Mucosal Drug Delivery and Mucosal Vaccination Strategies

FcRn is one of the few molecules known to move from the luminal to serosal membranes of the polarized cells that form the epithelial barriers of the lung and intestine (16, 43), thereby providing unique opportunities for potent non-invasive delivery of protein therapeutics across mucosal interfaces. This non-invasive mucosal drug delivery approach capitalizes upon two important prerequisites: (i) the knowledge that FcRn is functionally expressed throughout adult life in the upper and central airways as well as intestines of humans and non-human primates (8, 9, 44, 45) (ii) the knowledge that FcRn binds to the Fc portion of IgG in a pH-dependent manner and prevents degradation of monomeric ligand. Thus, when linked to an Fc fragment, therapeutic molecules benefit from both FcRn dependent half-life extension (15) and also mucosal absorption across epithelial boundaries.

The first study successfully demonstrating FcRn dependent delivery of an Fc containing therapeutic was performed in mice with a fusion protein between mouse erythropoietin (Epo) and the Fc fragment of mouse IgG1, showing that bioactive Fc-Epo fusion protein was successfully absorbed FcRn dependently via the respiratory epithelium (8). In subsequent studies in non-human primates, pulmonary delivery of a human Epo Fc-fusion protein was also successfully accomplished when the fusion proteins were aerosolized with a particle size of 4–6 mm to target the upper airways (44). In both mice and monkeys, the FcRn-dependence of this transepithelial transport was proven by the demonstration that an EpoFc-fusion containing the non-FcRn binding IHH variant of IgG was only poorly absorbed (44). In addition to that, it has already been demonstrated that this pathway is also operative in humans. A phase I clinical trial assessing the efficacy of the delivery of an Epo Fc-fusion protein into the bloodstream after administration of the fusion protein in the upper airways was able to show a dose-dependent uptake of the fusion protein with retained biologic activity (46). These studies laid the foundation for the broader possibility of transepithelial delivery of different types of Fc-fused macromolecular cargo including interferon-α, interferon-β, follicle-stimulating hormone, and nanoparticles (47–50). Thus, these studies demonstrate that FcRn-dependent drug delivery across mucosal surfaces is a novel non-invasive delivery approach that is both potent and clinically feasible.

Interestingly, exploitation of this mechanism is not necessarily restricted to the adult organism. Using a murine model of beta-glucuronidase (GUS) deficiency, in which glycosaminoglycan storage begins in prenatal life, it was shown that a GUS-Fc-fusion protein, infused into the mothers, was transported across the placenta into the fetus, leading to reduced lysomal storage disease only in those mice whose mothers were administered GUS-Fc (51). While this approach outline that FcRn binding to the Fc region of IgG might be exploited in severe cases for the administration of Fc-based treatment strategies starting *in utero*, these studies clearly imply that pathogenic antibodies can also cross the maternal–fetal barrier. That this is indeed the case has been shown in a murine model of fetal and neonatal immune thrombocytopenia (FNIT) in which FcRn-mediated transplacental transfer of maternal pathogenic antibodies results in the destruction of platelets in the neonate (52) but saturation of FcRn by intravenous infusion of IgG (IVIG) or blocking of FcRn in the mother prevented antibody-mediated FNIT (53). Apart from pathogenic antibodies, virion-IgG complexes, as demonstrated for cytomegalovirus, can disseminate in the placenta and gain access to the fetal circulation by co-opting the FcRn-mediated transplacental IgG transport pathway (54). Collectively, these studies demonstrate that the relevance of FcRn’s functions in the placenta extend far beyond the simple acquisition of passive immunity but also have broad implications for understanding neonatal (patho)immunology and the development of novel treatment strategies.

As shown recently, the interaction between FcRn and IgG can not only be co-opted for novel and non-invasive drug delivery approaches but also for the induction of humoral immunity along mucosal barriers. FcRn’s ability to confer humoral immunity is based on its capacity to bidirectionally transcytose IgG molecules across the epithelium lining mucosal barriers and preliminary studies have successfully demonstrated that FcRn conferred protective passive humoral immunity against infections at the site of pathogen entry when neutralizing antibodies were administered to the systemic circulation before infection (24, 29, 35). This was shown *in vivo* for both bacterial and viral agents, where FcRn-mediated translocation of systemically administered pathogen-specific IgG to the mucosal site of pathogen entry within the gastrointestinal or genital tract was identified as the key mechanism responsible for protection (24, 29, 35).

In addition to passive immunity, it has already been demonstrated *in vivo* that FcRn can also confer active humoral immunity. A prerequisite in these studies was that pathogens were, in order to enable FcRn-mediated transport of the antigen across the mucosal barrier, presented as Fc-fusion proteins and were administered in the presence of potent adjuvants to force an immune response. Intranasal immunization with a fusion protein between herpes simplex virus type-2 (HSV-2) glycoprotein gD and the Fc domain of IgG2a together with the adjuvant CpG, protected wild type, but not FcRn knockout, mice after intravaginal challenge with virulent HSV-2 and this immunization strategy induced an efficient mucosal and systemic antibody response as well B- and T-cell immunity (55). Similarly, mice that were intranasally vaccinated with a fusion protein between HIV Gag p24 and the Fc of mouse IgG2a together with CpG as an adjuvant developed local and systemic immunity, including durable B- and T-cell memory. In these experiments, Gag-specific immunity was sufficiently potent to protect against an intravaginal challenge with recombinant vaccine virus expressing the HIV Gag protein (56). Of particular importance, mice lacking FcRn or those immunized with free antigen or an antigen fused to an Fc fragment disabled in FcRn binding were not protected against HSV or HIV infection, thereby highlighting the central role of FcRn and its intact binding to IgG for efficient mucosal transport across an epithelial barrier and the elicitation of active mucosal immunization (55, 56). Finally, as shown in transgenic animals, overexpression of FcRn can significantly enhance humoral immunity by the following mechanisms: (i) prolonged IgG half-life, (ii) facilitation of antigen presentation by antigen-presenting cells (APCs), resulting in increased antigen-specific humoral immune response with larger numbers of antigen-specific B cells, and (iii) generation of antibodies against weakly immunogenic antigens, as recently reviewed elsewhere (57). Table 1 summarizes therapeutic applications and opportunities derived from FcRn-based biology.

**Table 1:** **Therapeutic applications and opportunities derived from FcRn-based biology**.

FcRn-based therapeutic approaches	Mode of action	Therapeutic use	Reference
**TRANSPORTING IgG**			
Respiratory epithelium	Transcytosis of IgG across respiratory epithelium	Non-invasive pulmonary delivery of IgG-based therapeutics in non-human primates and humans	(8, 44, 46–48)
		Vaccination against respiratory pathogens	
Intestinal epithelium	Transcytosis of IgG across intestinal epithelium	Providing passive and active immunity against enteric pathogens and infections	(31, 34, 35, 58)
	Transepithelial transport of Fc-targeted nanoparticles	Enabling drugs currently limited by low bioavailability to be efficiently delivered through oral administration	
Genitourinary epithelium	Transcytosis of IgG across genitourinary epithelium	Providing passive and active immunity against infections with genitourinary entrance	(55, 56)
Placenta	Transcytosis of IgG across the placenta from mother to fetus	Providing passive immunity to fetus	(51–53, 59–61)
		Transplacental delivery of IgG-based therapeutics	
**REGULATING IgG HALF-LIFE**			
Extending serum half-life of IgG and IgG-based therapeutics by augmenting FcRn interactions	Increasing FcRn binding with selective mutations within the IgG Fc fragment that enable pH-dependent binding of IgG	Increasing bioavailability of IgG- and IgG-based therapeutics in serum and tissue, effectiveness shown for	(42, 62, 63, 65, 64, 66)
		Prolonging factor VIII and IX serum half-life for hemophilia treatment	
		Enhanced anti-tumor activity	
		Protection against (S)HIV	
	Increasing FcRn binding to multimeric IgG containing immune complexes	Increasing and inducing MHC I and MHC II antigen presentation	(21, 31, 34, 36, 37, 67)
		Increasing and inducing antigen-specific T-cell responses	
		Relevance and effectiveness shown for	
		Tumor protection at mucosal sites (intestines, lung)	
		Vaccination with tumor antigen for increased anti-tumor surveillance	
Reducing serum half-life of IgG- and IgG-based therapeutics	IVIG effect and saturating FcRn binding	Amelioration of IgG-mediated diseases such as Immune thrombocytopenic purpura Myasthenia gravis	(15, 19, 68–78)
	Peptide mimetics or anti-FcRn antibodies that block IgG binding		
	Engineered antibodies with higher FcRn affinity that enhance IgG degradation (*Abdegs*)		
		Colitis	
		Arthritis	
		Pemphigus vulgaris	
		Autoimmune encephalomyelitis	
		Lupus nephritis	

## Concluding Remarks

In the recent past, the view of FcRn has changed from a receptor, which contributes to passive immunity via transcytosis of IgG molecules to that of an active signaling and trafficking receptor that is deeply involved in antigen presentation pathways. It is now clear that FcRn inherits a fundamental role in classical MHC II presentation of exogenous antigen as well in the cross-presentation of exogenous antigen on MHC I molecules. The pleiotropic functions enable FcRn to elicit antigen-specific CD4^+^ and CD8^+^ T-cell responses and thus exert broad functions in immune surveillance and immune homeostasis. Critically, these pathways have already been shown to have important pathophysiological relevance for infectious diseases and cancer development along mucosal barriers. Exploitation of the receptor–ligand interaction between FcRn and IgG has already been successfully used for the development of novel mucosal vaccination strategies as well as the non-invasive delivery of Fc-fusion proteins. Thus, the roles that FcRn plays in guiding the transport of IgG across epithelial barriers and regulating the recycling of monomeric IgG away from a degradative fate in lysosomes and preserving IgG half-life or alternatively directing IgG containing ICs to the antigen presentation machinery present enormous therapeutic opportunities for translation (Table 1). Our increasing understanding of the biology of FcRn will likely only increase the range of therapeutic applications for FcRn-targeted drugs, which promise to deliver effective treatment for neoplastic, infectious, and autoimmune diseases.

## Conflict of Interest Statement

The authors declare that the research was conducted in the absence of any commercial or financial relationships that could be construed as a potential conflict of interest.
